# A machine learning-based system for detecting leishmaniasis in microscopic images

**DOI:** 10.1186/s12879-022-07029-7

**Published:** 2022-01-12

**Authors:** Mojtaba Zare, Hossein Akbarialiabad, Hossein Parsaei, Qasem Asgari, Ali Alinejad, Mohammad Saleh Bahreini, Seyed Hossein Hosseini, Mohsen Ghofrani-Jahromi, Reza Shahriarirad, Yalda Amirmoezzi, Sepehr Shahriarirad, Ali Zeighami, Gholamreza Abdollahifard

**Affiliations:** 1 Shiraz University of Medical Sciences, Shiraz, Iran; 2 Department of Medical Physics and Engineering, School of Medicine, Shiraz University of Medical Sciences, Shiraz, Iran; 3 Shiraz Neuroscience Research Center, Shiraz University of Medical Sciences, Shiraz, Iran; 4 Department of Parasitology and Mycology, School of Medicine, Shiraz University of Medical Sciences, Shiraz, Iran; 5 Department of Medical Parasitology and Mycology, School of Medicine, Shiraz University of Medical Sciences, Shiraz, Iran; 6 Department of Pediatrics, Ilam University of Medical Sciences, Ilam, Iran; 7 Thoracic and Vascular Surgery Research Center, Shiraz University of Medical Sciences, Shiraz, Iran; 8 Department of Community Medicine, School of Medicine, Shiraz University of Medical Sciences, Shiraz, Iran; 9 Substance Abuse and Mental Health Research Center, Shiraz University of Medical Sciences, Shiraz, Iran

**Keywords:** Leishmania, Cutaneous leishmaniasis, Artificial intelligence, Image processing, Adaboost, Viola-Jones, Algorithm

## Abstract

**Background:**

Leishmaniasis, a disease caused by a protozoan, causes numerous deaths in humans each year. After malaria, leishmaniasis is known to be the deadliest parasitic disease globally. Direct visual detection of leishmania parasite through microscopy is the frequent method for diagnosis of this disease. However, this method is time-consuming and subject to errors. This study was aimed to develop an artificial intelligence-based algorithm for automatic diagnosis of leishmaniasis.

**Methods:**

We used the Viola-Jones algorithm to develop a leishmania parasite detection system. The algorithm includes three procedures: feature extraction, integral image creation, and classification. Haar-like features are used as features. An integral image was used to represent an abstract of the image that significantly speeds up the algorithm. The adaBoost technique was used to select the discriminate features and to train the classifier.

**Results:**

A 65% recall and 50% precision was concluded in the detection of macrophages infected with the leishmania parasite. Also, these numbers were 52% and 71%, respectively, related to amastigotes outside of macrophages.

**Conclusion:**

The developed system is accurate, fast, easy to use, and cost-effective. Therefore, artificial intelligence might be used as an alternative for the current leishmanial diagnosis methods.

## Background

Leishmaniasis, a disease caused by more than 20 species of leishmania parasites, is recognized in the tropical and subtropical regions as an acute disease with a high mortality rate. The disease manifests itself in both cutaneous and visceral forms and is transmitted via parasite-infected mosquitoes [1, 2]. Cutaneous Leishmaniasis (CL) is endemic in more than 88 countries and around two-third of the cases occur in Afghanistan, Algeria, Brazil, Pakistan, Peru, Saudi Arabia, Iran, and Syria [3, 4]. Annually, CL is estimated to cause 1 million new cases [5], with limited responsed in treatment and management [6–10].

The clinical symptoms of CL vary depending on the species of the parasite, but the disease, in general, begins with a papule or nodule, reaching its final size in about a week. Its center contains a shell that may break apart and show a wound that will heal slowly over months or years [11]. However, an estimated 10% of CL cases become chronic and progress into severe symptoms [12]. Considering the wide clinical spectrum of CL, certain diseases are likely to have similar clinical manifestations (e.g. dermatitis, squamous cell carcinoma, tuberculosis, skin mycosis). Therefore, to differentiate CL from its clinical and histologic look-alikes, additional diagnostic measures need to be taken [12].

Even today, parasitological diagnosis of CL remains the gold standard due to its high specificity [13]. The process includes a microscopic examination of biopsies with Giemsa stained or aspirates, histological examination of fixed lesion biopsies, culture, triturates, or aspirates biopsy [14]. Currently, microscopic examination is probably the most common diagnostic method because it is less expensive and available at the level of primary, secondary, and tertiary healthcare. Among these methods are protein A (ProtA), immunoglobulin (Ig)G2, lymphocyte proliferation assay, indirect fluorescent antibody test (IFAT), the quantitative real-time polymerase chain reaction of bone marrow (qPCR-BM), qPCR-Blood, and IgG [15]. Cultivation is another diagnostic method that provides useful information for the identification and description of species, but it is time-consuming and requires expenditures and technical expertise. Moreover, the sensitivity of this method is quite low [16].

Molecular parasitic diagnosis of CL has been extensively developed and reviewed over the past decade [17]. Diagnosis is mainly performed by PCR-based methods and is particularly useful in cases of low parasitic multiplicity (e.g., mucosal Leishmaniasis). Moreover, the treatment of CL patients can be controlled and followed up by this method. The specificity of this technique is 100, but its sensitivity is around 20% to 30% in CL and 55% to 70% in mucosal leishmaniasis which is low compared to conventional parasite detection methods. Several efforts have been made to improve the performance of molecular parasitic diagnosis of CL such as the successful discovery of parasitic DNA in blood or tissue stains; development of rapid PCR oligo-chromatography; however, its applications are still limited because this method is expensive and it also needs considerable laboratories’ infrastructure and technical expertise [17].

In this paper, we developed an artificial intelligence (AI)-based system to assist with detecting and diagnosing leishmania parasites. Details of the method and its evaluation results using several real images are presented.

## Methods

We used 300 images taken from 50 laboratory slides acquired from lesions suspected of leishmaniasis and from patients referred to Valfajr Clinic in Shiraz, Fars, Iran. These images include 150 photos from 25 positive leishmania slides and 150 photos from 25 negative Leishmania slides (control). The slides were prepared and labeled by taking samples from inflamed edges of the wounds using a sterile scalpel and smeared on slides, followed by 100% ethanol fixation and Giemsa staining.

According to the morphological data acquired by assessing the slides, the Viola-Jones algorithm was used to design an intelligent system capable of detecting infection in the collected smears. Briefly, the detector should be provided with images of both parasitic and non-parasitic samples so that it can gradually learn their distinctive features and become able to spot infected regions in an unseen image. Viola-Jones algorithm acts in the following steps: feature extraction, integral image creation, and classification [18].

**Fig. 1:**
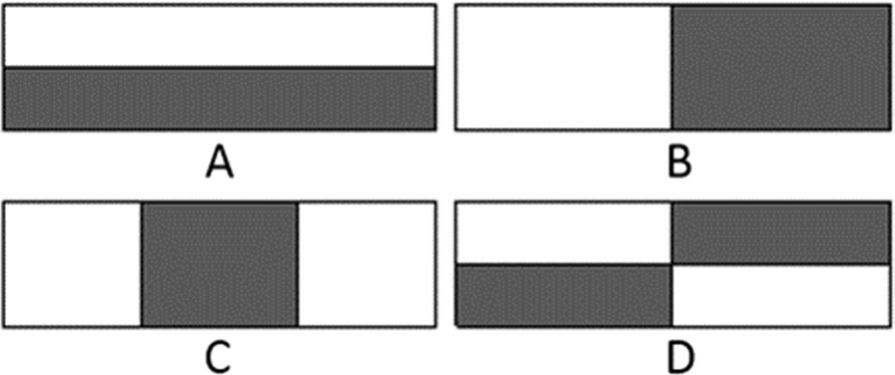
Rectangular window detection of Haar-like feature [19]

For feature extraction, the sum of the pixels within the white rectangles is subtracted from the sum of pixels in the grey rectangles (Fig. 1). The result is used as features to represent subsections of an image. Intuitively, these rectangle features are inspired by Haar wavelets which are simply square functions with various scales and translations.

Integral image creation was used to increase the processing speed and the number of features., The images used contain irrelevant parts. Moreover, an abundant number of Haar-like features should be computed. Particularly, 162,336 features were computed for a 24 × 24 pixel image window. To resolve this issue, we used the image integration technique by which the intensity of each pixel at $\left( {x,y} \right)$ is the sum of all the pixels that reside above and to the left side.

**Fig. 2:**
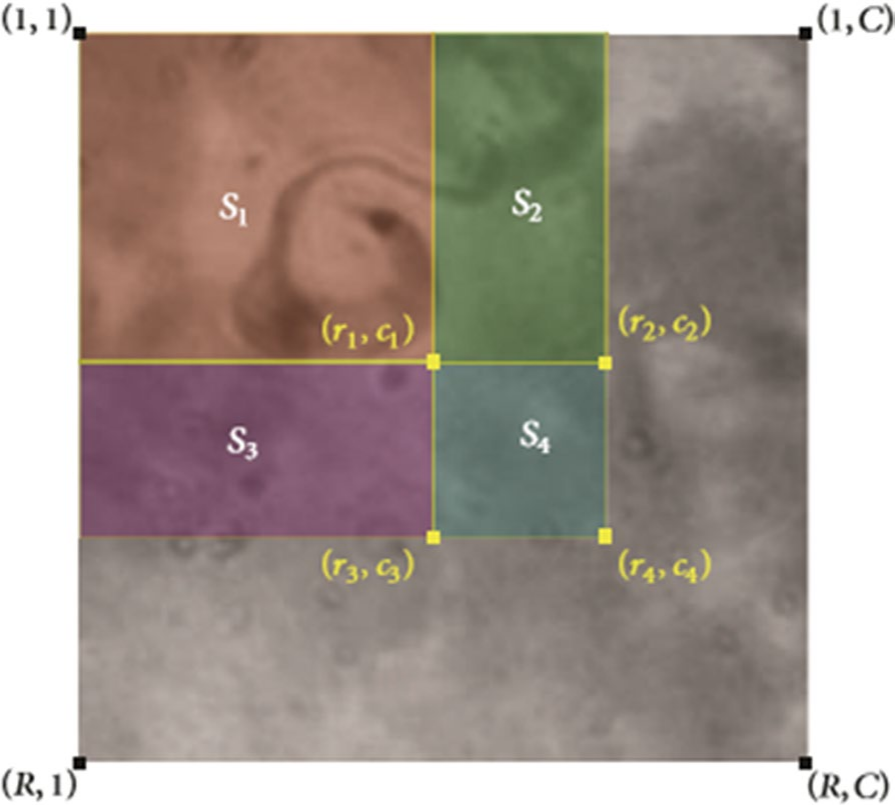
The complete expression of the integral image; the sum of the pixels inside sub-window S_1_ using the relation $\sigma \,\left( {S_{1} } \right) = ii\,\left( {p_{1} } \right) = ii\,\left( {r_{1} ,c_{1} } \right)$ is obtained and also the sub-windows S_2_ and S_3_ with the relations $\sigma \left( {S_{2} } \right) = ii\,\left( {r_{2} ,c_{2} } \right) - \left( {r_{1} ,c_{1} } \right)$ and $\sigma\, \left( {S_{3} } \right) = ii\,\left( {r_{3} ,c_{3} } \right) - ii\,\left( {r_{1} ,c_{1} } \right)$ are expressed. The pixels below S_4_ are also calculated as $\sigma\, \left( {S_{4} } \right) = ii\,\left( {r_{4} ,c_{4} } \right) - ii\,\left( {r_{3} ,c_{3} } \right) - ii\,\left( {r_{2} ,c_{2} } \right) + ii\,\left( {r_{1} ,c_{1} } \right)$. (Image by Uc-Cetina et al. [20])

By categorizing the subsections using Haar-like features, we can create integral images. The reason behind the categorization is to eliminate unwanted sections of our image and shorten processing time. To compute the sum of the pixel values in the subsections, array references are used. A single-rectangle sub-window needs four array references, while two, three, and four adjacent rectangle sub-windows need six, eight, and nine references, respectively. In an integral image of size $R \times C$, the main integral image $ii\left( {R,C} \right)$ is produced during single processing of the sum of the pixel values above and to the left of $\left( {R,C} \right)$. Once the integral image representation *ii *of the original image *I* is computed, the sum of original pixel values within any rectangle can be computed by a lookup table. Therefore, as shown in Fig. 2, to compute the sum of pixel values in subsection *S*_1_, (*r*_1_, *c*_1_) is needed and is computed as mentioned below:

$$\sigma \,\left( {S_{1} } \right) = ii\,\left( {r_{1} ,c_{1} } \right)$$

whereas to compute the values of subsection *S*_4_ reference arrays (*r*_1_, *c*_1_), (*r*_2_, *c*_2_), (*r*_3_, *c*_3_) and (*r*_4_, *c*_4_) are needed.

$$\sigma\, \left( {S_{4} } \right) = ii\,\left( {r_{4} ,c_{4} } \right) - ii\,\left( {r_{3} ,c_{3} } \right) - ii\,\left( {r_{2} ,c_{2} } \right) + ii\,\left( {r_{1} ,c_{1} } \right)$$

**Fig. 3:**
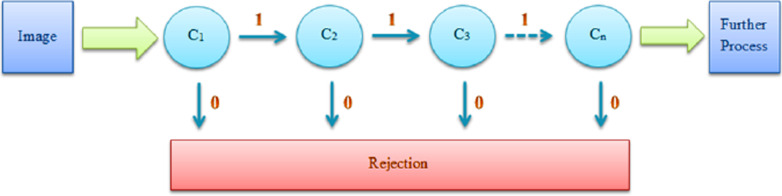
Adaptive boosting. The step-by-step process of tweaking classifiers

Therefore, aside from creating integral images, certain learning algorithms are employed to select the best features and to train classifiers. Adaboost, currently the most popular boosting method, acts by adding weak learners to a boosted classifier one by one. This way, each new classifier is trained using a new set of information. The resulting classifiers are integrated with a cascade scheme (Fig. 3).

Cascading is a stage-by-stage process, each stage consisting of a particular classifier with certain features. While all the features are grouped in these stages, the purpose of each stage is to determine whether a particular sub-window is not a match with the desired result or it may be a match; the desired result being the recognition of the previously defined morphological data. If a sub-window fails to find a match in any of the stages, it is discarded immediately. Therefore, usually, a classifier consisting of only a few simple and general features is used in the first stage/stages to rapidly remove unwanted subjects, granting more computational time to further stages requiring deeper analysis. Alternatively, a cascade of gradually more complex classifiers can achieve better detection rates, at the high cost of run-time speed, making it inefficient to do so. The sensitivity threshold can be adjusted in a cascade, preventing each stage from having a lower detection rate than the specified threshold. The total sensitivity will be the product of stage sensitivities. Ultimately, cascading classifiers enable the detection of the desired object in an image to be gradually approximated and a robust classifier is developed. Viola-Jones algorithm results in a drastic improvement of accuracy and execution time. In a given dataset $\left( {x_{1} ,y_{1} } \right),\left( {x_{2} ,y_{2} } \right), \ldots ,\left( {x_{n} ,y_{n} } \right),y_{i}$ is selected as 0 for negative cases and $y_{i} = 1$ for positives.

I. For, $y_{i} = 0$ *1* weights are considered to be $w_{1,j} = \frac{1}{2m},\frac{1}{2l}$, respectively; where *m* is the number of negative cases and *l* the number of positives.
II. For *t* = 1, …, *T*:
  - Weights are normalized as shown,
    $$\frac{{w_{t,i} }}{{\mathop \sum \nolimits_{j = 1}^{n} w_{t,j} }} \to w_{t,i}$$
  - Train the classifier *h*_*j*_ for each $jth$ feature, being restricted to using a single feature. The error is computed using the equation $w_{t} , \in_{j} = \mathop \sum \limits_{i} w_{i} \left| {h_{j} \left( {x_{i} } \right) - y_{i} } \right|$.
  - The classifier with the lowest ∈_*t*_ error, *h*_*t*_, is chosen.
  - The weights are updated as:
    $$w_{t + 1,i} = w_{t,i} \beta_{t}^{{1 - e_{i} }},$$
    *β*_*t*_ = ∈_*t*_/(1−ϵ_*t*_), and *e*_*i*_ = 0,1 for correct and incorrect classification of x_i_, respectively.
III. The strong classifier ensembled from single weak classifiers is as follows:
  $$h\left( x \right) = \left\{ {\begin{array}{*{20}c} 1 & {\mathop \sum \limits_{t = 1}^{T} \alpha_{t} h_{t} \left( x \right) \ge \frac{1}{2}\mathop \sum \limits_{t = 1}^{T} \alpha_{t} } \\ 0 & {otherwise} \\ \end{array} } \right.,$$
  $${\text{where }}\alpha_{t} = \log \left( {\frac{1}{{\beta_{t} }}} \right)$$

## Results

The performance of the developed system was quantitatively assessed using the two evaluation metrics sensitivity and specificity. Sensitivity, the probability of a positive test outcome in an infected patient, is calculated as shown below:

$${\text{Sensitivity}} = \frac{True \,positive}{{True\,positive + False \,negative}}$$

**Fig. 4:**
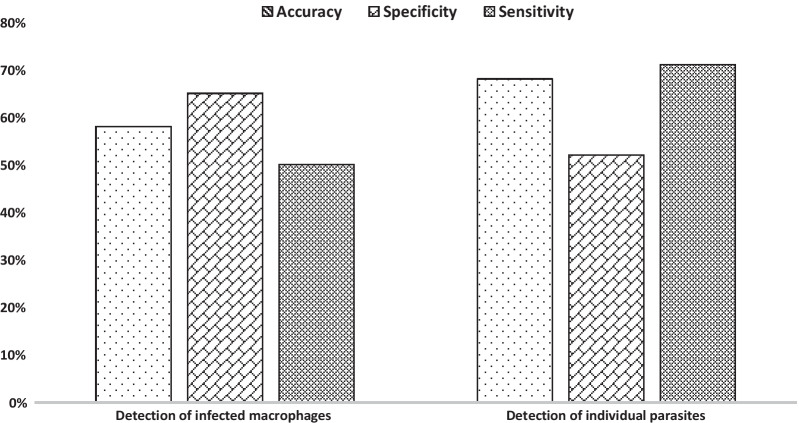
The accuracy, sensitivity, and specificity and of leishmania detection system, both in and outside of macrophages

Therefore, the fewer the number of false negatives, the higher the sensitivity will be. In this study, this rate was computed to be 50% and 71% for infected macrophages and amastigotes outside of macrophages, respectively (Fig. 4).

Additionally, the chance of a negative test in a healthy patient, known as specificity, can be calculated similarly;

$${\text{Specificity}} = \frac{True \,negative}{{True \,negative + False\, positive}}$$

Meaning that a low count of false positives increases the likelihood of the method is precise. Specificity in the detection of Leishmanial infected macrophages was shown to be 65%, while it was 52% for individual parasites (Fig. 4).

Overall, when the output of the infected macrophages-based system and individual parasites-based system were combined using OR combiner, the system provided a sensitivity and specificity of 83% and 35% in parasite detection, respectively.

## Discussion

In recent years, many methods have been suggested to diagnose the leishmanial parasite [21]. Each method was successful in several aspects, but there are several disadvantages associated with each method. Direct visual recognition using a microscope is a simple and cost-efficient method for parasite detection; however, it depends on the skillfulness of the expert and its sensitivity rate is relatively low [22]. Culture use, as another method for parasite detection, requires its own set of tools and expenses, and the probability of infection with other microbial organisms during the process might negatively affect the results [23]. Serological tests such as IFA and ELISA face the same issue as they cannot differentiate past and present infections. Additionally, serological tests, due to low antibody titers of the leishmanial parasite, do not offer much diagnostic value [24]. Early diagnosis of deadly diseases, such as leishmaniasis, results in an earlier treatment/control which can influence mortality rates significantly. Presently, PCR is known as the method presenting the highest sensitivity and specificity rates. Aviles et al. reported 92% sensitivity and 100% specificity in cutaneous leishmaniasis detection [24]. Similar results were obtained in many other studies [25–28]. However, in chronic cases, PCR sensitivity drops significantly (45.5%) [25]. In addition, PCR is a complex, expensive, and time-consuming procedure requiring certain devices. In this work, we examined the efficiency of artificial intelligence to detect leishmaniasis. Fortunately, the results were promising. The proposed system provided the sensitivity and specificities of 35% and 83% in detecting CL.

Many machine learning methods have been developed over the years which can help learning methods and diagnostic systems work more efficiently [29]. Adaboost, decision tree, KNN, linear regression, Naïve Bayes, Random Forest, and Extra tress are some of these methods. Saiprasath G et al. compared these 7 methods in an automated microscopic malaria detection procedure. The two methods, Random Forest and Adaboost proved to be more capable of generating desired results in terms of accuracy, sensitivity, specificity, and F1-score [30].

High recall rates are equivalent to a smaller count of false negatives. In deadly diseases such as Leishmaniasis, this percentage matters since infected patients should not be left unrecognized with the possibility of incorrectly being assumed healthy. Thus, necessary and deserved care and treatment can be provided, resulting in a lower morbidity and mortality rate. On the other hand, a high precision percentage indicates a low number of false positives. In some situations, the inadequacy of resources could prevent health experts from giving patients the care they need. A high number of false positives in a method could lead to unnecessary expenditure of resources and equipment and an increase in total expenses.

Bearing in mind the mentioned strengths and advantages of using intelligent diagnostic systems, keeping a heads-up in certain situations can help prevent any loss of efficiency. For example, if images acquired for the system contain low resolutions or have numerous dark parts (increased pixel count), the classification process would take more time, with the possibility of a greater number of false positives, thus overall efficacy drops. Moreover, these programs might need updates from time to time [31]. Thung et al. introduced Speeded-Up Robust Features (SURF)to develop an efficient method for automated detection of parasites. This procedure uses only images, without any need for learning and/or boosting algorithms. Unfortunately, the outcome was unsatisfactory [31]. Several procedures have been shown to perform based on Image Segmentation [32]. K-means clustering [33] and U-Net architecture are examples of the techniques used in this process. Górriz M et al. achieved promising results using U-Net architecture for Leishmanial parasite detection [33]. However, this method is quite time-consuming (15 h required by an NVIDIA GTX Titan x GPU) [33]. Nevertheless, this procedure can be performed considerably faster using integral image creation and boosting methods such as Adaboost [20].

## Conclusion

In this study, we proposed an AI-based system for cutaneous leishmaniasis detection. For this purpose, the Viola-Jones object detection algorithm enhanced by the Adaboost method was used. The system provided a fairly high sensitivity rate (83%), and moderate specificity rates. In addition, the algorithm is fast and easy to use. Overall, the results are promising and show that AI techniques can assist with diagnosing and treatment of leishmaniasis.

## Data Availability

The datasets used and analyzed during the current study are available from the corresponding author on reasonable request.

## Abbreviations

CL: Cutaneous leishmaniasis
SURF: Speeded-up robust features

## References

[1] Organization WH. Control of the leishmaniases: report of a meeting of the WHO Expert Commitee on the Control of Leishmaniases, Geneva, 22–26 March 2010: World Health Organization; 2010.

[2] Modabberi F, Ghadimi S, Shahriarirad R, Nadimi E, Karbalay-Doust S, Rashidi S (2021). Stereological analysis of liver, spleen and bone of Leishmania infantum-experimentally infected hamsters. Exp Parasitol.

[3] Meireles CB, Maia LC, Soares GC, Teodoro IPP, Gadelha MDSV, da Silva CGL (2017). Atypical presentations of cutaneous leishmaniasis: a systematic review. Acta Tropica..

[4] Norouzinezhad F, Ghaffari F, Norouzinejad A, Kaveh F, Gouya MM (2016). Cutaneous leishmaniasis in Iran: results from an epidemiological study in urban and rural provinces. Asian Pac J Trop Biomed.

[5] Ware JM, O’Connell EM, Brown T, Wetzler L, Talaat KR, Nutman TB (2021). Efficacy and tolerability of miltefosine in the treatment of cutaneous leishmaniasis. Clin Infect Dis.

[6] Ghadimi SN, Homayoon L, Shahriarirad R, Fatehpour S, Rastegarian M, Sarkari B (2019). Attenuated Leishmania major induce a high level of protection against Leishmania infantum in BALB/c mice. Iran J Parasitol.

[7] Sarkari B, Mohseni M, Moein MR, Shahriarirad R, Asgari Q (2017). Effect of hydroalcoholic extract of Echinacea purpurea in combination with meglumine antimoniate on treatment of Leishmania major-induced cutaneous leishmaniasis in BALB/c mice. Int J Appl Basic Med Res.

[8] Sarkari B, Sattari H, Moein MR, Tamadon AM, Rad RS, Asgari Q (2016). Effect of topical gel prepared with hydroalcoholic extract of Echinacea purpurea on treatment of Leishmania major-induced cutaneous leishmaniasis in BALB/C mice. J Pharm Negative Results.

[9] Pouryousef A, Eslami E, Shahriarirad S, Zoghi S, Emami M, Cheraghi MR (2021). Effects of topical gel formulation of Ficus carica latex on cutaneous leishmaniasis induced by Leishmania major in BALB/c mice. BMC Res Notes.

[10] Palumbo E (2009). Current treatment for cutaneous leishmaniasis: a review. Am J Ther.

[11] Hailu A, Dagne DA, Boelaert M (2016). Leishmaniasis. Neglected tropical diseases-Sub-Saharan Africa.

[12] Gurel MS, Tekin B, Uzun S (2020). Cutaneous leishmaniasis: a great imitator. Clin Dermatol.

[13] Mouttaki T, Morales-Yuste M, Merino-Espinosa G, Chiheb S, Fellah H, Martin-Sanchez J (2014). Molecular diagnosis of cutaneous leishmaniasis and identification of the causative Leishmania species in Morocco by using three PCR-based assays. Parasit Vectors.

[14] Escobar MA, Martinez F, Smith DS, Palma GI (1992). American cutaneous and mucocutaneous leishmaniasis (tegumentary): a diagnostic challenge. Tropical doctor..

[15] Rodríguez-Cortés A, Ojeda A, Francino O, López-Fuertes L, Timón M, Alberola J (2010). Leishmania infection: laboratory diagnosing in the absence of a “gold standard”. Am J Trop Med Hyg.

[16] Chargui N, Bastien P, Kallel K, Haouas N, Akrout FM, Masmoudi A (2005). Usefulness of PCR in the diagnosis of cutaneous leishmaniasis in Tunisia. Trans R Soc Trop Med Hyg.

[17] Reithinger R, Dujardin J-C (2007). Molecular diagnosis of leishmaniasis: current status and future applications. J Clin Microbiol.

[18] Viola P, Jones M, editors. Rapid object detection using a boosted cascade of simple features. Proceedings of the 2001 IEEE computer society conference on computer vision and pattern recognition CVPR 2001; New York: IEEE, 2001.

[19] Viola P, Jones MJ (2004). Robust real-time face detection. Int J Comput Vision.

[20] Uc-Cetina V, Brito-Loeza C, Ruiz-Piña H (2015). Chagas parasite detection in blood images using AdaBoost. Comput Math Methods Med..

[21] Akhoundi M, Downing T, Votýpka J, Kuhls K, Lukeš J, Cannet A (2017). Leishmania infections: molecular targets and diagnosis. Mol Aspects Med.

[22] Aronson NE, Joya CA (2019). Cutaneous leishmaniasis: updates in diagnosis and management. Infect Dis Clin.

[23] Weina PJ, Neafie RC, Wortmann G, Polhemus M, Aronson NE, Strausbaugh LJ (2004). Old world leishmaniasis: an emerging infection among deployed US military and civilian workers. Clin Infect Dis.

[24] Aviles H, Belli A, Armijos R, Monroy FP, Harris E (1999). PCR detection and identification of Leishmania parasites in clinical specimens in Ecuador: a comparison with classical diagnostic methods. J Parasitol..

[25] Vega-López F (2003). Diagnosis of cutaneous leishmaniasis. Curr Opin Infect Dis.

[26] Safaei A, Motazedian MH, Vasei M (2002). Polymerase chain reaction for diagnosis of cutaneous leishmaniasis in histologically positive, suspicious and negative skin biopsies. Dermatology.

[27] Ryan JR, Smithyman AM, Rajasekariah G-H, Hochberg L, Stiteler JM, Martin SK (2002). Enzyme-linked immunosorbent assay based on soluble promastigote antigen detects immunoglobulin M (IgM) and IgG antibodies in sera from cases of visceral and cutaneous leishmaniasis. J Clin Microbiol.

[28] Hailu A (2002). The use of direct agglutination test (DAT) in serological diagnosis of Ethiopian cutaneous leishmaniasis. Diagn Microbiol Infect Dis.

[29] Latif J, Xiao C, Imran A, Tu S, editors. Medical imaging using machine learning and deep learning algorithms: a review. 2019 2nd International Conference on Computing, Mathematics and Engineering Technologies (iCoMET); New York: IEEE; 2019.

[30] Saiprasath G, Babu N, ArunPriyan J, Vinayakumar R, Sowmya V, Soman K. Performance comparison of machine learning algorithms for malaria detection using microscopic images. IJRAR; 2019.

[31] Thung F, Suwardi IS, editors. Blood parasite identification using feature based recognition. Proceedings of the 2011 International Conference on Electrical Engineering and Informatics; New York: IEEE; 2011.

[32] Aimi Salihah A-N, Yusoff M, Zeehaida M. Colour image segmentation approach for detection of malaria parasites using various colour models and k-means clustering. 2013.

[33] Górriz M, Aparicio A, Raventós B, Vilaplana V, Sayrol E, López-Codina D, editors. Leishmaniasis parasite segmentation and classification using deep learning. International Conference on Articulated Motion and Deformable Objects; Springer: Cham. 2018.

